# Longitudinal links between maternal directives, children’s engagement in family conversations, and child linguistic skills

**DOI:** 10.3389/fpsyg.2023.1175084

**Published:** 2023-05-04

**Authors:** Tiia Tulviste, Anni Tamm

**Affiliations:** Department of Psychology, University of Tartu, Tartu, Estonia

**Keywords:** talk input, CDI-III, language comprehension, language production, directives, vocabulary diversity, children’s contribution

## Abstract

**Background:**

Research on mother–child verbal interaction is largely inspired by Vygotsky. The results align with his view that children acquire language and culture-specific ways of using language through actively participating in daily conversations with adults. Supporting Vygotsky’s concept of the Zone of Proximal Development, the facilitative features of such conversations have been found to depend on age, the level of the child’s language skills, and the interactional context. Most previous studies in the field have been conducted in English-speaking Western families with a focus on the first years of children’s lives. As Estonian middle-class mothers have been found to put greater emphasis on controlling children than mothers from other cultural contexts, we included the frequency of using directives as one of the features of mothers’ speech that might have an impact on child language development.

**Aim:**

Accordingly, the current study explored the relative impact of various aspects of mother–child interaction (e.g., mothers’ vocabulary diversity, use of attentional and behavioral directives, wh-questions, and the amount of children’s talk) on children’s language skills using data collected from Estonian middle-class families at two timepoints, 1 year apart. As a novel approach to this topic, the study also examined the correlation between mothers’ input features and children’s participation in the parent–child conversation.

**Method:**

A total of 87 children aged 3;0 and 4;0 and their mothers participated in the study. We observed the mother–child interactions during a semistructured videotaped game played at home. Mothers reported their children’s language skills *via* the ECDI-III. Children’s language comprehension and production were measured using the examiner-administered NRDLS.

**Results and conclusion:**

Although the results showed somewhat differential effects of various aspects of mothers’ speech on different measures of child language skills at two timepoints, the diversity of mothers’ speech was positively, and mothers’ frequent use of directives negatively related to children’s language skills. At both ages, the diversity of mothers’ speech predicted the amount of children’s verbal contribution to conversations. The findings will be discussed in light of Vygotskian and his followers’ theoretical views and theories about child language development.

## 1. Introduction

The current study assessed various features of mothers’ input during mother–child play interaction to determine which aspects of input have the greatest impact on children’s verbal participation in these conversations, both in the present and over time, as well as on their language skills at ages 3;0 and 4;0. Much research on child language acquisition and development has been inspired by, and the results are in line with, Vygotsky’s (1978) views that children acquire language and culture-specific ways of its use through active participation in early daily conversations with adults.

### 1.1. Facilitative features of parent speech

Although some variability in child language development is rooted in heritability (Stromswold, 2001), communicative exchanges with adults are important for children to acquire language. Since the book by Snow and Ferguson (1977) speech directed to language-learning children has received much scholarly attention. Studies have specified which kind of parent–child conversation plays a vital role in promoting child language skills. Much emphasis is placed on the quantity of adult speech (often measured as word tokens) directed at the language-learning child (Hart and Risley, 1995; Huttenlocher et al., 2010; Zauche et al., 2017), as well as on the quality of the language input, often measured as vocabulary diversity and sophistication (Huttenlocher et al., 2010; Rowe, 2012; Rowe and Snow, 2020). As Vygotsky (1978) proposed, children acquire the linguistic forms their parents use in daily conversations (Huttenlocher et al., 2010). Children who experience less verbal interaction face an increased risk of developing poorer language skills. Over the last few decades, studies have provided strong evidence that interactivity—engaging children in back-and-forth conversations—is the key feature that supports child language learning rather than being exposed to language (Romeo et al., 2018).

Supporting Vygotsky’s (1978) concept of the Zone of Proximal Development (ZPD), the facilitative features of adult input have been found to depend on children’s age and level of language skills (Rowe and Snow, 2020; Anderson et al., 2021). The language level of the child rather than the child’s age also determines how much parental speech is directed to children (Dailey and Bergelson, 2022). Moreover, research has revealed somewhat differential effects of various aspects of parental speech at different child ages. The amount of adult language input during the second year of life, the use of diverse and sophisticated vocabulary during the third year of life, and the use of decontextualized language such as narratives and explanations during the fourth year of life have been posited to play an important role in children’s future language skills (Huttenlocher et al., 2010; Rowe, 2012).

Additionally, wh-questions (e.g., who, what, when, where, and why questions) are found to be a useful type of input for fostering toddlers’ language learning (Rowe et al., 2017). Unlike other types of questions (e.g., yes-no questions), wh-questions allow children to provide more than one possible answer and elicit more verbal participation in conversations. Wh-questions are also linguistically and cognitively more challenging, as they elicit more syntactically complex responses (Rowe et al., 2017).

### 1.2. Mixed effects of directives

Not all features of parental talk that may relate to children’s language development have been assessed in previous studies. For example, the effect of parental use of directives on children’s early language learning is a less examined aspect of parent talk compared to features such as the number of word tokens, word types, and asking wh-questions. At the same time, parents differ in their communicative intent to control or converse with their children (McDonald and Pien, 1982; Hoff-Ginsberg, 1991; Tulviste, 2019a). Conversation-eliciting utterances are positively related to children’s language development, whereas developmental associations between different types of verbal control and language skills are not well known (Pine, 1992; Flynn and Masur, 2007; Rantalainen et al., 2022). In many studies, the directive conversational style of parents has been associated with children’s poorer language skills (Hampson and Nelson, 1993; Hart and Risley, 1995), but the direction of the effect remains unclear. Due to directives being easily understandable, parents use them to make their speech more easily understandable to children with poor language skills. Parental speech contains fewer directives when children become older and are more able to manage tasks independently (Pan et al., 1996; Tulviste, 2019a). Moreover, parents’ directiveness and functions have been found to differ across interactional contexts, being higher in toy play than in some other contexts (Hoff-Ginsberg, 1991; Yont et al., 2003).

Vygotsky (1978, 2012) explained how children acquire language and learn to contribute to conversations through participating in social interactions: social (i.e., interindividual) processes are the source of individual internal (i.e., intraindividual) processes (Wertsch, 1985). Unlike conversation-eliciting utterances such as wh-questions, directives may reduce a child’s engagement in adult–child conversations and, in turn, be linked to poorer language skills as they discourage children from practicing their language skills. Moreover, a high level of parental control might decrease children’s intrinsic motivation (Deci et al., 1993). Therefore, it could be that children of mothers who use directives more frequently participate less actively in conversations and, as a result, develop poorer language skills than children of mothers who frequently engage their children in conversations. By providing children the experience of talking more with the aim of conversing with their children rather than controlling their behavior or attention, parents can scaffold children’s ability to actively and verbally participate in conversations and engage others in conversations. Children can learn the pragmatic meaning of language from their mothers: is it a social tool to share information, or is it a tool to control and regulate other people’s attention and behavior? Similarly, it is likely that from early on, it is through everyday parent–child conversations at home that children implicitly learn how much talk is expected of them in conversations and how to participate in such conversations. It is likely that individual differences in talkativeness also have a genetic basis (Stromswold, 2001), but the quantity and quality of parental input also play a role in explaining the variability in how much children talk during conversations. Mothers who talk more tend to have more talkative children. It is known that parents talk more to infants who have just begun to talk and to toddlers with higher language skills (Dailey and Bergelson, 2022).

### 1.3. Cultural variation in parent–child communication

Despite a growing understanding of the relative importance of language-promoting features of talk, much of this knowledge is still derived from research conducted primarily on English-speaking Western, mostly American, middle-class families (Tulviste, 2019b). Little is known about the extent to which the facilitative features of parent talk are culture-specific. However, evidence from other cultural and linguistic contexts is necessary to check the generalizability of previous findings. A wide cultural range exists in family interaction patterns: how common adults’ one-to-one dyadic conversations with children are, the number of words children hear, how directive parents are, and how much children are involved in conversations (see Tulviste, 2019b). Differences in the use of language in children with diverse cultural backgrounds have been attributed to the culture-specific patterns of such conversations. There are likely some cultural differences in what features of mother–child interaction foster a child’s language learning the most. For example, it is unclear which input features should support children in cultures where talkativeness is generally less highly valued and control of children is more highly valued.

Furthermore, there is considerable evidence that some aspects of parental input and children’s language development are linked to the child’s gender and maternal SES. Girls have been found to have better language skills than boys of the same age (Fenson et al., 2007; Eriksson et al., 2012). There are conflicting results about whether parents speak differently to girls versus boys (Leaper and Smith, 2004). According to some studies, girls receive larger language input and hear more questions and repetitions, but fewer directives and attention-getters, than boys (Clearfield and Nelson, 2006). Similarly, an Estonian study with LENA-generated estimates found that 4;0 old girls heard more speech than boys. However, the boys’ environment was noisier (Tulviste and Tamm, 2021). Other studies have reported no differences in the quantity and quality of parental speech based on a child’s gender (see Leaper and Smith, 2004; Rantalainen et al., 2022).

Parents with higher levels of education (a core component of SES) have been found to engage in speech patterns that more strongly promote children’s language development. They have been found to talk more with their children and use a greater variety of words with fewer directives (Heath, 1983; Hart and Risley, 1995; Hoff, 2006; Fernald et al., 2013; Rowe, 2018). SES-related differences in children’s language development have already been found among 18-month-old children (Fernald et al., 2013).

Thus, the crucial role of parental talk in children’s language learning is consistently validated in studies. To better understand the mechanisms underlying language development, studies with more features of parent talk that may matter are still needed to provide more information about which specific features of adult–child interaction are developmentally appropriate for children of different ages and in children with other cultural backgrounds.

### 1.4. The present study

The current study aimed to determine the extent to which different aspects of maternal speech (vocabulary diversity, the frequency of using attentional and behavioral directives, and wh-questions) contribute to children’s language skills and conversational contributions to mother–child play interactions at children ages 3;0 and 4;0 years.

As a novel approach to this topic, we focused on the extent to which the features of mothers’ talk facilitate children’s language skills and their contribution to conversations, i.e., to how talkative the children are. The diversity of mothers’ vocabulary was calculated as the number of produced word types per minute and children’s verbal contribution to conversations—talkativeness—as tokens per minute told by children. These two spontaneous speech measures (i.e., types and tokens) were derived from the same mother–child conversation during a joint toy play. It is known from prior studies that tokens and types are strongly associated. Talkative parents tend to produce speech that is more varied in terms of vocabulary. Considering that parents’ vocabulary diversity rather than input quantity has been found to predict children’s language learning in toddlers and preschoolers (Rowe, 2012; Anderson et al., 2021), we focused on the use of different words by mothers.

Unlike many other studies on this topic, parental attentional and behavioral directives were included as features of mothers’ talk that might affect concurrent and future language development. To our knowledge, to date, no studies have directly examined the relative importance of maternal directiveness for children’s language skills. Estonian middle-class mothers of toddlers and teenagers tend to use more directives and fewer conversation-eliciting utterances than, for example, Swedish and US mothers (Junefelt and Tulviste, 1997; Tulviste et al., 2003). Moreover, directives in the form of imperatives are very common in the Estonian language, and the frequent use of imperatives when speaking in Estonian is not perceived to be as unpolite as it is, for example, in Swedish, especially when directives are mitigated by adding “please” or “honey” (Metslang, 2004). Despite greater parental verbal control and other peculiarities of the Estonian language, Estonian children do not differ from children with other cultural backgrounds in linguistic skills (Eriksson et al., 2012; Kuvać-Kraljević et al., 2021). Estonian parents’ conversational intent to frequently control children’s attention and behavior might be reflected in the finding that Estonian 4-year-old children were less active conversation partners during the past event talk than their Swedish counterparts, as they answered their mothers’ questions rather than spoke on their own initiative (Tulviste et al., 2016). Thus, in the current study, we included more items (the frequency of using attentional and behavioral directives) among the measures of mothers’ input talk than previously, addressing not only children’s language skills as outcome measures but also their contribution to conversations.

Children’s language skills were measured using two assessment tools. Language comprehension and production were assessed using the examiner-administered standardized New Reynell Developmental Language Scales (NRDLS, Edwards et al., 2011). Similar to most prior studies, we also used parental reports of a child’s communicative abilities—the Estonian version of the CDI III (E-CDI-III, Tulviste and Schults, 2020). Most research to date has focused on the effects of the quality and quantity of parent talk on variability in children’s vocabulary during the first 3 years of life. The present study was conducted with 3–4-year-old children. At this age, not only the variety of words but also the acquisition of other aspects of language, such as the variety of syntactic structures, pronunciation, etc., are good indicators of language acquisition. Hence, we focused on more general language abilities and used the total score of the E-CDI III instead of the vocabulary score. The assessment tools used in the study allowed us to examine how differences in mothers’ input reflect in children’s language knowledge (i.e., comprehension scores) and language use (i.e., language production scores and the parent-reported E-CDI-III total score).

This study aimed to examine the relationship between various aspects of mothers’ input and children’s language skills and participation in conversation at two time points when the children were 3;0 and 4;0 years old. According to Vygotsky (1978), scaffolding (i.e., regulating children’s behavior and providing guidance and feedback) in the ZPD—the split between the actual skill level and the level that the child achieves with the help of the adult’s guidance and regulation—is highly relevant when children are learning a new skill. Scaffolding decreases as children’s skills develop, and they are gradually more and more able to perform tasks independently (Vygotsky, 1978). At age 3;0, children begin to play collaboratively with other people and become more verbally involved in mother–child interactions. Thus, it is likely that mothers’ input differs at two time points. At age 3;0, children might require more regulations from their mothers *via* directives for joint play with toys.

Similarly, their need for mothers’ wh-questions for engaging in mother-child joint play interaction might be higher 1 year later when these skills have grown. Moreover, children’s language and conversational skills are higher at age 4;0. Thus, attentional and behavioral directives might have a greater negative effect, while wh-questions have a greater positive effect at age 3;0 compared to age 4;0.

Another aim of this study was to investigate the longitudinal links between mothers’ input features in children aged 3;0 and child outcomes at 4;0 years. By relying on Vygotskian theory (Vygotsky, 1978), we hypothesized that children whose parents elicited conversations from them more frequently by asking wh-questions learned the importance of their verbal participation in family conversations. As they age by 1 year, these children are more communicative and show higher language and conversational skills than children whose mothers asked fewer questions and were more concerned with regulating their attention or behavior through directives. While exploring the predictors of children’s developmental outcomes, we were also interested in whether the findings hold even after controlling for the same skills 1 year earlier.

The research questions of the study are as follows:

1. How similar are the predictors of children’s language skills and contribution to conversations at two-time points—at age 3;0 and 4;0—when controlling for the child’s age and maternal education?
2. To what extent are children’s language skills at Wave 2 predicted by the features of mothers’ talk input and children’s contribution to conversations at Wave 1, controlling for the child’s previous language skills, gender, and mother’s education?

## 2. Materials and methods

### 2.1. Participants

The sample consisted of 105 mother-child dyads at Wave 1 and 87 dyads at Wave 2. The children were, on average, 3;0 years old (*M*age = 35.77 months, SD = 0.84 months; 60 females and 45 males) at Wave 1 and 4;0 years old (*M*age = 48.31, SD = 0.61 months) at Wave 2. The inclusion criteria for the study required that children be around 36 months old, that both mothers and children be Estonian speakers, and that the children have no serious health or language problems. For maternal education, there were two categories: those with at least a university education, i.e., a bachelor’s degree (58%), and those with less than a university education (42%).

### 2.2. Procedure

The children’s families were first contacted close to the children’s third birthday. Children were video recorded in their homes in semistructured toy play interactions with their mothers. The mothers were given a bag containing kitchen and doctor toys and asked to play with their children as they normally would. No time restrictions were set for their joint play. A research assistant administered the NRDLS to each child during the next visit to the children’s home. At Wave 1, we collected the family’s background information.

### 2.3. Measures

Mother–child play interactions were transcribed using the Computerized Language Analysis (CLAN) program (MacWhinney, 2000). *Word tokens* (i.e., the number of words used by the child) and *word types* (i.e., the number of different words used by the mother) were taken from the automated computer analyses of the transcripts by the CHAN program. Because the length of the play sessions differed, we used the number of word types per minute to measure mothers’ diversity of vocabulary and the number of word tokens per minute as a measure of children’s contribution to conversations, i.e., their talkativeness.

#### 2.3.1. The coding of play interactions

We coded mothers’ directives and wh-questions. All directives—utterances used to give verbal directives to the child—were identified and divided into two categories depending on whether the mother aimed to control the children’s attention or behavior. The utterances that involved giving commands or permission, requesting or encouraging desirable action, or preventing the child from acting (e.g., “*Put the cup on the table!*”) were categorized as behavioral directives. The utterances used to get the child’s attention (e.g., *Listen carefully*!” and “*Look, I put it here*!”) or calling the toddler’s name (e.g., “*Marleen*!”) were categorized as attentional directives. The wh-questions referred to the questions beginning with who, what, when, where, and why (e.g., “*What should we do this morning?*” and “*What happened to her?*”). All repetitions were coded.

Mother–child interaction transcripts were coded by a research assistant. Another research assistant coded 20% of the transcripts to assess interrater reliability. The inter-rater reliability was measured by Kappa and ranged from 0.81 to 0.91.

#### 2.3.2. Children’s linguistic skills

##### 2.3.2.1. Estonian CDI-III

The ECDI-III (Tulviste and Schults, 2020) is the Estonian adaptation of the CDI-III developed for Swedish by Eriksson (2017) and consists of (1) *the level of communication* – a general evaluation of a child’s language complexity (max = 6); (2) *a 100-item vocabulary* list that contains food words, body words, mental words, and emotion words. For each word, the parent was asked to indicate whether the child says the word (max = 100); (3) *ECDI-III scores for grammar* consist of grammar usage and sentence complexity sections. The grammar usage section asks parents to indicate for seven items whether the child has never used it, used it several times, or used it on a daily basis (max = 34). The sentence complexity section contained 10 pairs of sentences, including simple and complex sentences. The parent was asked to indicate for each pair if the child currently uses the simple one, alternates between simple and complex ones, or uses a more complex one (max = 20); (4) *the metalinguistic awareness section* (phonological and orthographic awareness, max = 7); and (5) *the pronunciation section* (max = 7). The scores of all subscales were summed (max = 154).

##### 2.3.2.2. The New Reynell Developmental Language Scales (Edwards et al., 2011)

We used the most recent version of the well-known language test—the Reynell Developmental Language Scales—to assess the child’s comprehension and production of single words (nouns and verbs) and simple and complex sentences. The comprehension scale of the NRDLS consists of 72 items, and the production scale consists of 64 items. In the Estonian version, there is the same number of items as in the original English version, but some items in the pronouns, complex sentences, and grammatical judgment sections have been changed because the Estonian language differs from English. The norming of the Estonian version of the NRDLS has not been finished (and published). There are preliminary norms for 3–4-year-old children based on 255 children aged 34–50 months. In the present study, Cronbach’s alpha was used to assess the internal consistency of the items within the scales. These were 0.93 for the comprehension scale and 0.96 for the production scale. There was a high correlation between the two scales; *r* = 0.772 at age 3;0 and *r* = 0.868 at age 4;0.

To estimate the relative importance of variables pointed out in previous studies as central in predicting variability in children’s early language skills, we used generalized linear models. We used children’s gender, mothers’ education, vocabulary diversity (i.e., word types per minute), frequency of using attentional and behavioral directives, and wh-questions as predictors of children’s concurrent linguistic skills (i.e., CDI total score, comprehension, production, and talkativeness). The child’s talkativeness (i.e., tokens per minute) was added as a predictor in models where CDI total score, comprehension, and production were the dependent variables. Two spontaneous maternal speech measures—word tokens and word types—were strongly associated with each other (*r* = 0.794, *p* < 0.001, at Wave 1, and *r* = 0.746, *p* < 0.001 at Wave 2). Moreover, prior research has also shown that during the third year of life, the diversity of input starts to play a larger role than the amount of input (Rowe, 2012). When investigating longitudinal predictors of children’s language skills, we added the same language skills measured 1 year earlier among the predictors outlined earlier.

## 3. Results

### 3.1. Descriptive statistics

The mean scores for the study variables at two waves when children were around 3;0 and 4;0 are presented in Table 1. As shown in Table 1, the frequency of wh-questions asked by mothers was the only measure of interest that did not differ between the two waves. In comparison with Wave 1, the frequency of mothers’ use of behavioral directives decreased significantly (*p* < 0.0001), whereas the frequency of producing attentional directives increased (*p* < 0.01), and the diversity of mothers’ vocabulary increased (*p* < 0.0001) at Wave 2. Children’s talkativeness (i.e., tokens produced per minute) during play interaction increased (*p* < 0.05), as well as all three scores of their linguistic skills (*p* < 0.0001). At Wave 1, boys had lower production scores and contributed less to conversations than girls. At Wave 2, mothers used behavioral directives more frequently with boys. Children of more educated mothers scored higher on all language measures at both waves (except the E-CDI III Total score at Wave 2). They contributed more to conversations, and their mothers’ vocabulary diversity was greater than that of children whose mothers had lower levels of education.

**TABLE 1:** Descriptive statistics: means and standard deviations of the mother–child interaction variables and children’s language skills.

	Wave 1		Wave 2			
	***M***	**SD**	***M***	**SD**	***p***	**Cohen’s *d***
**Child**						
CDI total	79.35	26.32	110.07	20.92	<0.0001	-1.87
Comprehension	49.31	11.02	58.07	12.34	<0.0001	-0.72
Production	32.45	11.02	58.05	12.34	<0.0001	-1.07
Talkativeness	18.76	8.84	21.73	10.80	0.013	-0.27
**Mother**						
Behavioural directives	2.14	1.15	0.98	0.62	<0.0001	1.02
Attentional directives	1.43	0.64	1.86	1.38	0.004	-0.32
Wh-questions	2.57	1.54	2.64	1.42	ns	-0.06
Vocabulary diversity	19.86	6.36	22.38	5.94	<0.0001	-0.43

ns, insignificant differences between Wave 1 and Wave 2 scores according to paired samples t-tests. Means and standard deviations of all interaction variables are per min.

Tables 2, 3 present concurrent predictors and Table 4 presents longitudinal predictors of children’s language comprehension and production, CDI total score, and talkativeness.

**TABLE 2:** Predictors of children’s language skills and talkativeness at Wave 1.

	CDI		Comprehension		Production		Talkativeness	
	***B* (SE)**	***p***	***B* (SE)**	***p***	***B* (SE)**	***p***	***B* (SE)**	***p***
**Children**								
Boys (ref. girls)	0.84 (5.16)	ns	−0.45 (1.93)	ns	−6.18 (2.52)	0.014	−1.66 (1.61)	ns
Talkativeness	0.67 (0.33)	0.045	0.13 (0.13)	ns	0.14 (0.16)	ns	−	
**Mothers**								
Lower education (ref. higher)	−6.70 (5.75)	ns	−3.05 (2.14)	ns	−6.64 (2.78)	0.017	−5.33 (1.75)	0.002
Behavioral directiveness	−7.00 (2.69)	0.009	−3.48 (1.17)	<0.001	−4.43 (1.34)	<0.001	−1.65 (0.81)	0.041
Attentional directiveness	−3.59 (4.65)	ns	0.81 (1.74)	ns	2.34 (2.27)	ns	−2.35 (1.41)	ns
Wh-questions	0.90 (1.65)	ns	−0.46 (0.60)	ns	0.21 (0.79)	ns	−0.75 (0.51)	ns
Vocabulary diversity	1.66 (0.49)	<0.001	0.79(0.19)	<0.001	0.94(0.25)	<0.001	0.42(0.15)	0.004
Pearson *x*^2^/*df*	1.10		1.10		1.10		1.08	

**TABLE 3:** Predictors of children’s language skills and talkativeness at Wave 2.

	CDI		Comprehension		Production		Talkativeness	
	***B* (SE)**	***p***	***B* (SE)**	***p***	***B* (SE)**	***p***	***B* (SE)**	***p***
**Children**								
Boys (ref. girls)	−1.90 (4.78)	ns	−3.16 (1.64)	ns	−6.23 (2.31)	0.007	4.02 (2.32)	ns
Talkativeness	−0.09 (0.23)	ns	−0.05 (0.08)	ns	0.02 (0.11)	ns	−	
**Mothers**								
Lower education (ref. higher)	−1.60 (4.83)	ns	−4.55 (1.69)	0.007	−4.39 (2.38)	ns	−3.08 (2.41)	ns
Behavioral directives	−8.92 (3.82)	0.019	−1.26 (1.36)	ns	−1.08 (1.91)	ns	−3.15 (1.91)	ns
Attentional directives	−6.20 (1.64)	<0.001	−2.33 (0.59)	<0.001	−3.17 (0.83)	<0.001	−1.24 (0.83)	ns
Wh-questions	3.01 (1.56)	ns	0.45 (0.56)	ns	1.66 (0.78)	0.034	−1.42 (0.78)	ns
Vocabulary diversity	1.10 (0.43)	0.010	0.55 (0.15)	<0.001	0.57(0.21)	0.006	0.54 (0.20)	0.008
Pearson *x*^2^/*df*	1.13		1.11		1.11		1.09	

**TABLE 4:** Longitudinal predictors of children’s language skills and talkativeness at Wave 2.

	CDI W2		Comprehension W2		Production W2		Talkativeness W2	
	***B* (SE)**	***p***	***B* (SE)**	***p***	***B* (SE)**	***p***	***B* (SE)**	***p***
**Model 1**								
**Children**								
Boys (ref. girls)	−1.13 (4.51)	ns	−0.14 (2.52)	ns	−3.25 (2.60)	ns	3.72 (2.39)	ns
Talkativeness W1	0.34 (0.29)	ns	0.07 (0.17)	ns	0.04 (0.17)	ns	−	
**Mothers**								
Lower education (ref. higher)	−1.55 (5.03)	ns	−8.08 (2.81)	0.004	−5.97 (2.90)	0.039	−3.88 (2.64)	ns
Behavioral directives W1	−9.09 (2.28)	<0.001	−3.46 (1.27)	0.006	−4.78 (1.30)	<0.001	−1.66 (1.17)	ns
Attentional directives W1	−0.61 (4.01)	ns	1.34 (2.23)	ns	−0.07 (2.30)	ns	0.43 (2.07)	ns
Wh-questions W1	2.14 (1.34)	ns	0.18 (0.78)	ns	0.75 (0.81)	ns	−0.80 (0.75)	ns
Vocabulary diversity W1	1.01 (0.41)	0.014	0.37 (0.23)	ns	0.84 (0.24)	<0.001	0.29(0.21)	ns
Pearson *x*^2^/*df*	1.13		1.11		1.11		1.09	
**Model 2**								
**Children**								
Boys (ref. girls)	3.14 (3.06)	ns	1.03 (2.53)	ns	−0.64 (2.54)	ns	4.76 (2.29)	0.038
Talkativeness W1	−0.07 (0.20)	ns	−0.01 (0.18)	ns	0.01 (0.17)	ns	0.45 (0.15)	0.003
CDI W1	0.52 (0.07)	<0.001						
Comprehension W1			0.37 (0.14)	0.007				
Production W1					0.31 (0.10)	0.002		
**Mothers**								
Lower education (ref. higher)	2.20 (3.36)	ns	−7.15 (2.78)	0.010	−3.85 (2.80)	ns	−1.45 (2.63)	ns
Behavioral directives W1	−4.67 (1.69)	0.006	−2.11 (1.46)	ns	−3.02 (1.41)	0.032	−0.85 (1.14)	ns
Attentional directives W1	−0.29 (2.77)	ns	0.48 (2.29)	ns	−0.91 (2.26)	ns	1.73 (2.01)	ns
Wh- questions W1	0.20 (0.91)	ns	0.30 (0.77)	ns	0.43 (0.76)	ns	−0.62 (0.71)	ns
Vocabulary diversity W1	0.22 (0.30)	ns	0.09 (0.27)	ns	0.52 (0.26)	0.041	0.11 (0.21)	ns
Pearson *x*^2^/*df*	1.15		1.13		1.13		1.11	

#### 3.1.1. Concurrent predictors of child outcome measures

At Wave 1, CDI total scores were positively related to children’s talkativeness, mothers’ vocabulary diversity, and less frequent use of behavioral directives. Higher scores on comprehension scales were associated with mothers’ larger vocabulary diversity and less frequent use of behavioral directives. Higher scores on the production scale were related to being a girl, mothers’ higher education, and bigger vocabulary diversity. Children’s talkativeness was related to higher maternal education, a larger vocabulary diversity, and the less frequent use of behavioral directives.

At Wave 2, CDI total scores were positively related to the diversity of mothers’ vocabulary and the less frequent use of behavioral and attentional directives. Comprehension scores were positively related to mothers’ higher education, diversity of vocabulary, and less frequent use of attentional directives. Production scores were positively related to being a girl, mothers’ more frequent use of wh-questions, a bigger diversity of vocabulary, and less frequent use of attentional directives. Talkativeness was positively related to a larger diversity of mothers’ vocabulary.

### 3.2. Longitudinal predictors of children’s outcome measures

It is evident from Table 4 that the child’s E-CDI III Total Score at 4;0 years was predicted by the mothers’ use of a more diverse vocabulary and a lower frequency of using behavioral directives 1 year earlier. When we included the E-CDI III Total Score at 3;0 years in the model, only the CDI Total Score and the lower frequency of using behavioral directives remained as significant predictors of the E-CDI III Total score at 4;0 years.

The language comprehension score at 4;0 years was predicted by mothers’ higher education levels and less frequent use of behavioral directives at 3;0 years. When controlling for the comprehension score at 3;0 years, only the previous comprehension score and less frequent use of behavioral directives remained significant predictors.

The production score at 4;0 years was predicted by mothers’ vocabulary diversity and less frequent use of behavioral directives a year earlier. As shown in Table 4, both remained significant predictors when the language production score from a year earlier was controlled for.

None of the variables of interest predicted children’s talkativeness at 4;0 years. After controlling for children’s talkativeness a year earlier, this and being a girl were significant predictors.

Thus, the frequency of using behavioral directives and vocabulary diversity at 3;0 years were the most important aspects of mothers’ input that related to the child’s subsequent language skills.

## 4. Discussion

The study examined concurrent and longitudinal associations between the features of the mother’s talk input, children’s verbal contributions to play interaction, and their language skills.

Using the data collected at two-time points 1 year apart at 3;0 and 4;0 years, we found a significant increase in all children’s language skills that were measured, as well as in their verbal contributions to play interactions. Changes were also observable in mothers’ ways of talking with children (except in the frequency of asking wh-questions). The finding that mothers’ vocabulary diversity and children’s language skills significantly increased during one year is congruent with studies reporting that parents use more diverse language with language-advanced children (Dailey and Bergelson, 2022). The results also indicated that mothers directed children’s behavior significantly less and attention significantly more at children aged 4;0 years than they did 1 year earlier. There was some support for previous studies suggesting that girls have better language skills than boys and that parents converse differently with girls and boys (Leaper and Smith, 2004; Clearfield and Nelson, 2006; Fenson et al., 2007; Eriksson et al., 2012). Namely, girls scored higher on the language production scale, and their verbal contribution to conversations was bigger at age 3;0. Mothers used behavioral directives more frequently with boys at age 4;0. Mothers’ education was linked to many of the variables central to the study, despite the relatively high educational level of the mothers participating in our study. Specifically, children of more educated mothers received higher scores on all language measures (except the E-CDI III Total score at Wave 2), they contributed more to conversations, and their mothers’ vocabulary diversity was greater than that of children with lower-educated mothers. Thus, the findings are in line with many previous studies (see Hoff, 2006).

### 4.1. Concurrent predictors of developmental outcomes at ages 3;0 and 4;0

Previous studies have pointed to the age-specificity of features of parent input that matter the most in early language development (Huttenlocher et al., 2010; Rowe, 2012). Based on these studies, we compared the predictors of children’s outcomes at two-time points. For children aged 3;0, mothers’ higher education predicted their children’s greater production scores and talkativeness, whereas at age 4;0, their higher comprehension scores. None of the outcome measures were predicted by the frequency of asking wh-questions by the mothers, except language production scores at Wave 2. At the same time, the mothers’ speech with their children, which contained a more varied vocabulary, was a significant predictor of all language scores at both waves and the children’s verbal contribution to play interaction.

An important finding of our study was that the frequent use of behavior directives was a significant negative predictor of all four outcome measures at Wave 1 and continued to be negatively linked to mother-reported language scores at Wave 2. At age 4;0, the frequent use of attentional directives predicted poor outcomes (except for children’s talkativeness). Thus, the study sheds light on the different roles of maternal attentional and behavioral directives in a child’s language development at different times. Interpreting the findings in light of the Vygotskian theory of ZPD (Vygotsky, 1978), a reason that behavioral directives play such a big negative role in predicting younger children’s language skills might be the 3-year-olds’ limited abilities of cooperative play. Moreover, the toys provided by the experimenter were new to them, and likely because of that, more behavioral directives were required to guide the children’s play activities. Mothers’ use of behavioral directives twice as much with younger children supports the presumption. A year later, children seemed to need mothers’ directives to keep their attentional focus on ongoing play rather than guidance on how to play with their mothers and new toys.

Similarly, a lack of change in the frequency of asking wh-questions may indicate that as children become older and more communicative, they do not need mothers’ encouragement through wh-questions to engage in conversations. The findings that wh-questions did not predict the amount of talk contributed by children at 3;0 and 4;0 did not support our suggestion. It is worth noting that at Wave 1, the amount of children’s contribution to conversations did predict their parent-reported language skills but not the scores on the standardized test. It is possible that more talkative children look like they have better language skills, and mothers tend to overestimate their skills. On the contrary, it may also be that talkative children have good language skills, and their mothers’ estimates on the E-CDI-III are more accurate as they know better which words and grammatical constructs children already produce. Moreover, children who talk more with their mothers might be less talkative with an unknown research assistant who is administering the test. As a result, their language skills are underestimated by the standardized assessment.

At age 3;0, children’s talkativeness was predicted by their mothers’ higher education level, larger vocabulary diversity, and lower frequency of using behavior directives. At age 4;0, only vocabulary diversity mattered. Language comprehension scores at age 3;0 were predicted by mothers’ vocabulary diversity and a lower frequency of using behavioral directives. At age 4, language comprehension scores were predicted by mothers’ higher educational level, greater vocabulary diversity and less frequent use of behavioral directives. Language production scores at age 3;0 were predicted by being a girl, having a mother with a higher educational level, having a more diverse vocabulary, and using behavioral directives less frequently. At age 4;0 being a girl, and mothers’ diverse vocabulary, a lower frequency of using attentional directives, and asking wh-questions were significant predictors of productive language scores. Mother-reported language skills at Wave 1 were predicted by being more talkative and mothers’ vocabulary diversity and reduced use of behavioral directives, and at Wave 2, by mothers’ vocabulary diversity and reduced use of attentional and behavioral directives.

Thus, although the results showed somewhat differential effects of various aspects of mothers’ speech and background factors on the child’s concurrent language skills at two-time points, all concurrent language scores were positively predicted by mothers’ vocabulary diversity and negatively predicted by mothers’ frequent use of directives. The predictors of concurrent outcomes at two waves differed with regard to which directives mattered: a higher frequency of mothers’ behavioral directives was a negative predictor at age 3;0, whereas a higher frequency of attentional directives at age 4;0.

### 4.2. Longitudinal predictors of developmental outcomes

The second objective of the study was to investigate to what extent children’s language skills at Wave 2 were predicted by the features of mothers’ talk input and children’s talkativeness measured 1 year earlier, controlling for children’s gender and mothers’ education. We were also interested in whether the findings held when the same language scores from 1 year earlier were entered as predictors. The results indicated that mother-reported language scores and language production scores on the standardized test at age 4;0 were positively predicted by mothers’ earlier vocabulary diversity and negatively predicted by their earlier use of behavioral directives. When controlling for the same language scores 1 year earlier, the pattern of the results remained the same in the model that predicted language production. Mothers’ vocabulary diversity at age 3;0 did not remain a significant predictor of later mother-reported language skills when controlling for the same language scores 1 year earlier. Better language comprehension scores at age 4;0 were predicted by the mother’s higher education and less frequent use of behavioral directives 1 year earlier. When previous comprehension scores were counted, only these scores and mothers’ education remained significant predictors.

Given that plenty of recent research on child language acquisition highlights the importance of children’s verbal participation in conversations, we were interested in finding out which features of earlier input predict a bigger verbal contribution to mother-child play interactions 1 year later. We expected that parental control of the child’s behavior by means of directives would likely lead children to concentrate on play activities rather than verbal exchanges and, thus, not support language development. At the same time, one might think that mothers’ use of open-ended questions facilitates children’s ability to verbally participate in conversations and results in better language skills over time. Our longitudinal results showed that none of the variables measured 1 year earlier mattered in predicting how talkative children were at age 4;0. When statistically controlling for children’s earlier talk production, only these scores and being a girl were significant predictors. It is likely that talkativeness is more heritable than language skills and is less affected by the features of talk input.

### 4.3. Factors that matter the most in future language and conversational skills

The study addressed the question of which variables measured at age 3;0 affect children’s subsequent language skills the most. Our study advances the literature by revealing that all earlier outcome measures central to the study (i.e., mother-reported language scores and directly measured language comprehension and production scores) were important predictors of the same developmental outcomes measured 1 year later. The findings correspond to the results of many previous studies showing that language development during the first years of a child’s life plays a crucial role in later language proficiency (Hart and Risley, 1995; Rescorla, 2009; Golinkoff et al., 2019). After statistically controlling for the same outcome measure 1 year earlier, many other predictors did not remain significant. For example, although the frequency of using behavioral directives a year earlier was a significant negative predictor of all subsequent language outcomes central to the study, after controlling for the same language scores at Wave 1, they remained a significant predictor of language production scores and mother-reported language skills (except language comprehension scores). However, the finding is consistent with those of studies indicating that the frequent use of directives is a risk factor in language development (see Hoff, 2006).

In contrast, vocabulary diversity in maternal input at Wave 1 related positively to future language production scores and mother-reported language skills. However, when controlling for the same language skills at Wave 1, vocabulary diversity remained a significant predictor of later production scores. Various words have been proven to be a crucial feature of parental talk input that matters in a child’s language development during the third year of life (Rowe, 2012). Our findings are consistent with those of previous studies and add to the literature regarding the beneficial effects of vocabulary diversity on language learning also in slightly older children—around their third and fourth birthdays.

Based on recent theoretical views that children’s active participation in social interactions supports their language development, one might expect that talkativeness promotes the development of language skills over time. Our data did not support the presumption, as talkativeness did not emerge as a predictor of any future language skills. Wh-questions are commonly assumed to be related to children’s better language skills (Rowe et al., 2017). There was no support for the idea that children whose mothers promoted 3-year-old children’s conversations by more frequent use of wh-questions were more talkative 1 year later or that they had better language skills. Longitudinal analysis indicated that gender was a significant predictor only for children’s talkativeness and only when children’s previous contribution to the conversation was counted for. The results also did not confirm that mothers’ education is a significant predictor of subsequent language and communication abilities (Fernald et al., 2013). Education-related differences did appear only in future comprehension scores, and mothers’ educational level remained an important predictor of language comprehension even after controlling for previous scores on the comprehension scale.

### 4.4. Limitations of the study

A limitation is that the study was done with a constrained age range of children and only in Estonia. Researchers working in line with Vygotskian ideas pay a lot of attention to the developmental context in which children grow up. It is known that parents of different cultural backgrounds vary greatly in how they talk with their children, and Estonian middle-class mothers have put much more emphasis on verbal control of their children. In families with other cultural backgrounds where fewer directives are used during play interactions, negative associations between mothers’ directives and children’s concurrent and future language skills might be not as strong. The current study focused only on mothers’ input during play interaction, but the daily social context of children at ages 3;0 and 4;0 includes many different conversational partners, including fathers and other family members, kindergarten teachers, and other people outside the family. Research in other cultural and linguistic contexts and across various interaction contexts is necessary to check the generalizability of our findings. It is unclear if the language predictors that matter the most during the studied period are the same when children grow older.

## 5. Conclusion

Despite these limitations, the study advanced previous studies in the field. Most prior studies have addressed vocabulary development in infants and toddlers. The current study with slightly older children and its focus on more general language skills added to the literature the knowledge that each language skill of interest (i.e., mother-reported language skills, language comprehension, and production measured using a standardized language test), as well as children’s contribution to conversations, has a somewhat different combination of predictors. However, mothers’ diverse vocabulary is a positive predictor of concurrent and future language skills, whereas the frequent use of directives is a risk factor for language development. The results support the view that the features that foster children’s language learning the most depend on concrete developmental outcomes and the age/language skills of the child.

## Data availability statement

The raw data supporting the conclusions of this article will be made available by the authors, without undue reservation.

## Ethics statement

The studies involving human participants were reviewed and approved by the Research Ethics Committee of the University of Tartu. Written informed consent to participate in this study was provided by the participants’ legal guardian/next of kin.

## Author contributions

TT wrote the manuscript and made substantial contributions to the conception and design of the work. TT and AT interpreted the results, and organized and participated in data collection. AT carried out the statistical data analyses and made the language correction. Both authors provided approval for publication of the content and agreed to be accountable for all aspects of the work in ensuring that questions related to the accuracy or integrity of any part of the work are appropriately investigated and resolved.
